# Ultrasensitive Immunosensor Array for TNF-α Detection in Artificial Saliva using Polymer-Coated Magnetic Microparticles onto Screen-Printed Gold Electrode

**DOI:** 10.3390/s19030692

**Published:** 2019-02-08

**Authors:** Lassaad Barhoumi, Francesca G. Bellagambi, Federico M. Vivaldi, Abdoullatif Baraket, Yohann Clément, Nadia Zine, Mounir Ben Ali, Abdelhamid Elaissari, Abdelhamid Errachid

**Affiliations:** 1 NANOMISENE Lab, LR16CRMN01, Centre for Research on Microelectronics and Nanotechnology of Sousse, Technopole of Sousse B.P. 334, Sahloul 4034, Sousse, Tunisia; barhoumilassaad@yahoo.fr (L.B.); mounirbenali@yahoo.com (M.B.A.); 2 University of Sousse, Higher Institute of Applied Sciences and Technology of Sousse, GREENS-ISSAT, Cité Ettafala, Ibn Khaldoun 4003, Sousse, Tunisia; 3 Department of Chemistry and Industrial Chemistry, University of Pisa, via Giuseppe Moruzzi 13, 56124 Pisa, Italy; francesca.bellagambi@dcci.unipi.it (F.G.B.); federicomaria.vivaldi@phd.unipi.it (F.M.V.); 4 Université de Lyon 1, Institut des Sciences Analytiques, UMR 5280, CNRS, 5 rue de la Doua, 69100 Villeurbanne, France; a.baraket@gmail.com (A.B.); yohann.clement@isa-lyon.fr (Y.C.); nadia.zine@univ-lyon1.fr (N.Z.); abdelhamid.errachid@univ-lyon1.fr (A.E.); 5 Univ Lyon, University Claude Bernard Lyon-1, CNRS, LAGEP-UMR 5007, F69622 Lyon, France

**Keywords:** magnetic microparticles, immunosensor, tumor necrosis factor-α, chronoamperometry, saliva analysis, heart failure

## Abstract

Tumor necrosis factor-α (TNF-α) is a biomarker of inflammation that occurs in patients suffering from heart failure (HF). Saliva can be sampled in a non-invasive way, and it is currently gaining importance as matrix alternative to blood in diagnostic and therapy monitoring. This work presents the development of an immunosensor array based on eight screen-printed gold electrodes to detect TNF-α in saliva samples. Two different functionalization strategies of electrodes were compared. In the first, anti-TNF-α antibodies were chemically bonded onto the electrode by functionalization with 4-carboxymethylaniline. The other functionalization procedure involved the binding of antibodies onto polymer-coated magnetic microparticles, which were then deposited onto the electrode by pulsed chronoamperometry. Finally, the chronoamperometry technique was applied to characterize the modified SPEAu. The use of a secondary antibody anti-TNF-α (Ab-TNF-α-HRP) labelled with horseradish peroxidase (HRP, 2 µg·mL^−1^) was investigated using tetramethylbenzidine (TMB, pH = 3.75) as electrochemical substrate containing 0.2 mM of H_2_O_2_. A sandwich-type detection strategy with a secondary antibody anti-TNF-α provided chronoamperometric analyses in 10 s for each sample. Linearity, precision, limit of detection, and selectivity of devices were investigated. Interferences were evaluated by analyzing solutions containing other cytokine produced during the acute stage of inflammation. The immunosensor showed good performance within the clinically relevant concentration range, with a precision of 8%, and a limit of detection of 0.3 pg/mL. Therefore, it may represent a promising tool for monitoring HF in a non-invasive way.

## 1. Introduction

Tumor necrosis factor-α (TNF-α) is a pro-inflammatory cytokine, considered as a central mediator of a broad range of biological activities, especially in immune and inflammatory related diseases whose severity may be determined by TNF-α improper balance [1]. Dysregulation of TNF-α is correlated to various human diseases such as cancer [2], psoriasis [3], Alzheimer’s [4], major depression [5], and inflammatory bowel diseases [6]. Raised circulating levels of inflammatory cytokines, such as TNF-α, are also reported in patients suffering from heart failure (HF), with increasing levels according to disease severity [7,8,9]. The main cause of mortality and poor quality of life in Western societies [10], HF is a cardiovascular chronic disease caused by structural or functional abnormalities of the heart that make it unable to fill or to pump out blood, resulting in lower delivery of oxygen. Traditionally, the evaluation of HF patients is based on clinical assessment only. In recent years, the interest of cardiologists has been extended to biomarkers for prompt diagnosis and non-invasive HF monitoring, both important goals in Healthcare research.

Compared to blood or urine, saliva is a relatively simpler matrix that can be used for disease monitoring due to its non-invasive sampling from people at any age and even by multiple collections, as well as for the stability of several analytes in this medium [11,12,13,14].

TNF-α is usually quantified in saliva by enzyme-linked immunosorbent assay (ELISA) [15,16,17]. However, possible cross-reactions with other cytokines may occur and then affect the quality and validity of the immunoassay [18].

This paper describes the fabrication of a novel immunosensor array based on a screen-printed gold electrode (SPEAu) for the detection of the TNF-α in saliva samples. Two different SPEAu functionalization strategies were developed and compared: the first with a two dimensional (2D) SPEAu with anti-human TNF-α antibody (Ab-TNF-α) directly attached onto the electrode surface, and the second with a three-dimensional (3D) SPEAu with Ab-TNF-α linked to a new structure of magnetite magnetic microparticles (MMPs) coated with poly(pyrrole-co-pyrrole-2-carboxylic acid) (Py/Py-COOH) previously attached onto the SPEAu by pulsed chronoamperometry (PCA). Py/Py-COOH/MNPs were used to increase the surface are, and thus the number of antibodies, as the innovative aspect of this work. Antibody Ab-TNF-α was covalently bonded to Py/Py-COOH/MNP-modified gold WEs through amide bonding. The prepared immunosensor has been successfully applied to increase the sensitivity of Ag-TNF-α antigen in complex matrices such as saliva. Both SPEAu electrodes were designed as a substrate for a “sandwich-type assay” by using a secondary antibody anti-TNF-α labelled with horseradish peroxidase (Ab-TNF-α-HRP) and by employing tetramethylbenzidine (TMB) as electrochemical substrate. TNF-α standard solutions and artificial saliva samples spiked with different TNF-α concentration have been analyzed to test the final device. The immunosensor specificity was proven by analyzing different standard solutions containing other molecules, such as interleukin-6 (IL-6) and interleukin-10 (IL-10), that may represent possible salivary interference due to their production during acute stage of inflammation [19].

## 2. Experimental Section

### 2.1. Reagents and Solutions

Sodium chloride (NaCl, purity ≥ 99%), sodium nitrite (NaNO_2_, purity ≥ 97.0%), N-hydroxysuccinimide (NHS, purity 98%), 4-aminophenylacetic acid (CMA, purity 98%), and N-(3-dimethylaminopropyl)-N′-ethyl-carbodiimide hydrochloride (EDC-HCl, purity 98%), pyrrole (Py, purity 98%), povidone (PVP), pyrrole-2-carboxylic acid (Py-COOH, purity 99%), iron chloride hexahydrate (FeCl_3_⋅6H_2_O, purity 97%), purchased from Acros Organics (distributed by Thermo Fisher Scientific, Illkirch Cedex, France). Potassium chloride (KCl, purity 99%), phosphate buffer solution (PBS) tablets, sodium phosphate dibasic (Na_2_HPO_4_, PharmaGrade), anhydrous calcium chloride (CaCl_2_, purity ≥ 96.0%), potassium hexacyanoferrate (III) (K_3_Fe(CN)_6_, purity 99%), potassium hexacyanoferrate (II) trihydrate (K_4_Fe(CN)_6_⋅3H_2_O, purity ≥ 99.95%), pure ethanol, mucin, urea, interleukin 6 (IL-6), and interleukin 10 (IL-10) were purchased from Sigma Aldrich (Saint-Quentin-Fallavier, France). Sodium nitrite (NaNO_2_) has been purchased from Merck, whereas hydrogen peroxide (H_2_O_2_), sulfuric acid (H_2_SO_4_), and ethanolamine were from Fluka (Saint-Quentin-Fallavier, France).

MMPs oil-in-water magnetic emulsion (ME, total solid content 7.9%) has been acquired from Ademtech S.A. (Pessac, France). The image was processed for grain size measurement using ImageJ software. The average grain size is 1.07 μm.

Ab-TNF-α, Ab-TNF-α-HRP, TNF-α, and tetramethylbenzidine (TMB) were purchased from Abcam (Paris, France). Millipore Milli-Q nanopure water (resistivity > 18 MΩ cm) was produced by a Millipore Reagent Water System (Molsheim, France). SPEAus (250BT) have been purchased from Metrohm (Villebon-sur-Yvette, France).

### 2.2. Electrochemical Measurements

The 16 channels VMP3 potentiostat (BioLogic, France) controlled by EC-Lab software was used to carry out all the measurements at room temperature (20 ± 2 °C) in a Faraday cage. Auxiliary electrode (platinum) and reference electrode (Ag/AgCl) are designed to be used in flow injection analysis obtaining inlet and output flow perpendicular to the working electrodes surface, and amperometric measurements were performed in a flow-cell for 8X format SPEAu working electrode. Eight rubber seals delimit the volume of the eight independent electrochemical cells. Cyclic voltammetry (CV) measurements have been performed to investigate the optimum reduction potential for TMB substrate, to functionalized SPEAu electrodes and to characterize the SPEAu after CMA deposition.

### 2.3. Coating of Magnetic Microparticles with Poly (pyrrole-co-pyrrole-2-carboxylic Acid)

MNPs coated with poly (pyrrole-co-pyrrole-2-carboxylic acid) (Py/Py-COOH/MNPs) were obtained by the seeded polymerization technique described by Tenório-Neto et al. [20] and Hassani et al. [21]. Briefly, using a glass reactor, 1.25 g of ME (0.12 g of dried extract) were added and after 5 min, the supernatant was recovered. Then, 10 mL of aqueous solution containing 20 mg of PVP was then added in to the reactor. The emulsion was re-dispersed using a Teflon stirrer at 300 rpm for 4 h. The last step required the addition of 18 mg of Py-COOH (16 mmol), 8.5 μL of Py (16 mmol) and 90 mg of FeCl_3_·6H_2_O into the reactor, and the whole emulsion was kept under stirring for 12 h at room temperature (20 ± 2 °C).

### 2.4. Preparation of 2D-SPEAu

Ab-TNF-α and Ab-TNF-α-HRP were reconstituted in PBS buffer (10 mM, pH 7.4) according to the protocol provided by the supplier. Stock solutions of 125 µg·mL^−1^ Ab-TNF-α, and 33.5 µg·mL^−1^ Ab-TNF-α-HRP were then obtained by dilution. To promote the adhesion of the antibodies, CMA molecules has been electrochemically deposited onto SPEAu using CV in a 10 mM CMA aqueous solution containing 20 mM sodium nitrite. The potential was scanned from 0 V to –1.4 V vs. Ag/AgCl and five cycles were performed at a scan rate of 80 mV s^−1^. In order to confirm the electrodeposition, CV analysis was performed on both bare and functionalized electrodes by immersing the device into a 5 mM K_3_[Fe(CN)_6_]/K_4_[Fe(CN)_6_] in a PBS (pH 7.4) solution as redox couple. The voltammogram was recorded with a scan rate of 80 mV·s^−1^ and the potential was scanned between –0.4 to 0.6 V. In order to immobilize the Ab-TNF-α onto the electrode surface, carboxylic groups of the CMA molecules have been activated using 0.4 M EDC/0.1 M NHS coupling in ethanol solution for 1 h at room temperature (20 ± 2 °C). The device was then washed with HCl 0.1 M to remove the excess of EDC/NHS and then incubated for 1h at 4 °C in a 100 µg·mL^−1^ stock solution containing Ab-TNF-α (see Section 2.7). Finally, any activated group remaining on the surface was deactivated by incubation in ethanolamine solution (0.001% in PBS).

### 2.5. Preparation of 3D-SPEAu

**Figure 1:**
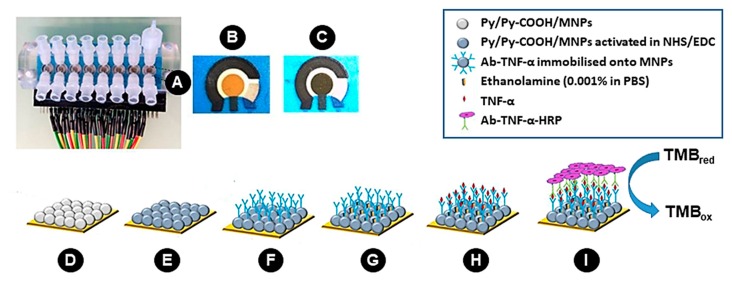
Schematic representation of preparation and detection strategy of our immunosensor. (**A**) Flow-cells for 8X format; (**B**) Stereo-microscopic image of bare SPEAu (gold surface); (**C**) Stereo-microscopic image of SPEAu modified by the electrodeposition of Py/Py-COOH/MNPs; (**D**) drawn of Py/Py-COOH/MNPs electrodeposited onto the SPEAu; (**E**) Py/Py-COOH/MNPs activated by incubation in ethanolic solution of EDC/NHS; (**F**) Ab-TNF-α (primary antibody) immobilized onto the MNPs surface; (**G**) Free active carboxylic groups deactivated by ethanolamine solution; (**H**) Antigen (TNF-α) capture by Ab-TNF-α; (**I**) TNF-α capture by Ab-TNF-α-HRP (secondary antibody).

This preparation was performed by electrodepositing Py/Py-COOH/MMPs onto SPEAu by using pulsed chronoamperometric technique (PCA). Briefly, two continuous repetition potentials have been applied: the first potential E_1_ = −1.5 V for t_1_ = 0.5 s, induces a cathodic electrodeposition process, while the second potential E_2_ = 1.5 V for t_2_ = 0.5 s oxidizes the electrode surface, and in particular, cleans it from eventual extraneous deposited species. The cycling process was performed 10 times. Then, the SPEAu were rinsed with distilled water. It is interesting to note that the amplitude of the charge-discharge process remains practically unchanged during the prolonged cycling process, indicating that the electrode surface exhibits a homogenous Py/Py-COOH/MNPs deposition. The activation of carboxylic groups present in the Py/Py COOH/MMPs was carried out by the mixture of EDC/NHS (0.4 M/0.1 M) prepared, as described above. The blocking step was performed by incubating the immunosensor in an ethanolamine solution/PBS (0.01% V/V). Finally, the immunosensor was rinsed with PBS and used for the detection of TNF-α (Figure 1C). The homogeneity and the roughness of the surface obtained were realized by three-dimensional topography of the surface by atomic force microscopy (AFM) and Electronique scanning microscope (SEM). The advantage of the use of these techniques is to guarantee the homogeneity of the surfaces in WEs with the presence of spherical shapes indicating the presence of MMPs coated with Py/Py-COOH.

### 2.6. Electrode Surface and Microparticles Characterization

Atomic force microscopy (AFM) was used to characterize the surface of 3D-SPEAu covered with Py/Py-COOH/MNPs. The analysis was performed using a Nano observer microscope (Concept Scientific Instruments, France) with a maximum area of scan is 110 μm^2^. The cantilever had a length of 125 μm, a width of 35 μm and a thickness of 4.5 μm. The tip radius was lower than 10 nm and the tip height (H) = 14.16 μm, with a frequency ranged between 200−400 kHz and a tip strength of K= 25.75 N·m^−1^. The scanning images were performed at 5 V amplitude, set point at 8 V and tip direct current at 477 nV. Measurements were carried out in a ‘tapping-mode’ (TM-AFM) which was used with a speed of 0.5 line·s^−1^ and a resolution of 1024 lines.

The morphology of Py/Py-COOH/MNPs electrodeposited onto the SPEAu was studied by scanning electron microscopy (SEM), performed using a JSM-5400 (JEOL, USA).

### 2.7. Standard Solutions and Sample Analysis

TNF-α have been reconstituted in PBS buffer (10 mM, pH 7.4) according to the protocol provided by the supplier, and a stock solution of 100 µg·mL^−1^ has been then obtained by dilution. The latter was then further diluted in PBS to have standard solutions with 1, 5, 10 and 15 pg·mL^−1^ of analyte.

Stock and standard solutions of IL-6 and IL-10 were prepared with the same procedure and at the same concentrations to investigate the immunosensor specificity.

Artificial saliva (AS) was prepared according to a procedure previously described [22,23]. The stock solution of TNF-α was diluted in AS to obtain samples at different concentration level (1, 5, 10 and 15 pg·mL^−1^) and to spike three aliquots of AS to obtain samples with known concentration (5 pg·mL^−1^) of TNF-α, successively used to simulate a real saliva sample analysis by performing the standard addition method.

The different samples of TNF-α in AS at different concentration levels were analyzed to investigate the linearity of the immunosensor. The same samples were also used to determine the precision and the limit of detection (LoD). The precision of the devices was determined by analyzing three replicate for each concentration level using a single device for each analysis. Eight AS samples containing 1 pg·mL^−1^ of TNF-α were analysed and the LoD was calculated in accordance with IUPAC guide-lines [24], as three times the standard deviation (s_b_) obtained on these samples and then divided by the sensitivity of the method (slope of the calibration function).

The immunosensor selectivity was investigated by analyzing standard solutions containing TNF-α and other solutions containing other cytokine produced during acute stage of inflammation such as Interleukin-6 (IL-6) and interleukin-10 (IL-10).

To perform the standard addition method, a constant volume (10 µL) of spiked AS was added to each of five volumetric flasks of 1 mL. Then, 990 µL of PBS (pH 7.4) were added to the first flask to reach a final volume of 1 mL. Increasing volumes (50, 150, 350, and 500 µL) of the TNF-α stock solution were subsequently added to the other flasks. Each sample was then made up to volume (1 mL) with PBS.

All the standard solutions and samples were prepared by weighing.

To measure TNF-α, 50 µL of sample were deposited onto the SPEAu and then the immunosensor was incubated for 30 min at 4 °C. After PBS washing, the conjugate Ab-TNF-α-HRP (50 µL, 2 μg·mL^−1^) was deposed onto the SPEAu, and the immunosensor was incubated again (30 min at 4 °C). After rinsing five times with PBS, the PCA response was recorded by immersing the immunosensor in the TMB solution. Each measurement was performed in triplicate.

## 3. Results and Discussion

### 3.1. Electrode Surface and Microparticles Characterization

**Figure 2:**
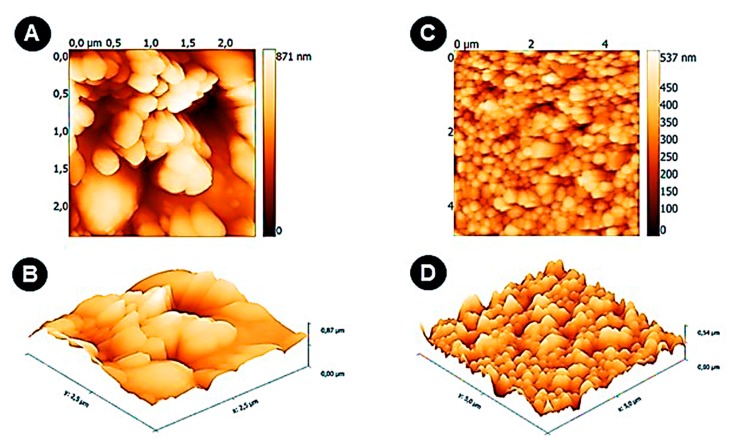
AFM (2D and 3D) topographic images of SPEAu surface: (**A**) the surface of bare SPEAu in 2D; (**B**) the surface of bare SPEAu in 3D. The dimensions of the image in X and Y coordinates are in the range of 2.5 μm × 2.5 μm; (**C**) SPEAu after the electrodeposition of Py/Py-COOH/MNPs (2D); (**D**) SPEAu after the electrodeposition of Py/Py-COOH/MNPs (3D). The dimensions of the image in X and Y coordinates are in the range of 5 μm × 5 μm.

Figure 2 shows 2D and 3D surface topography of both bare SPEAu (Figure 2A,B, respectively) and Py/Py-COOH/MNPs film after electrodeposition onto the SPEAu (Figure 2C,D, respectively).

The bare SPEAu showed a homogeneous surface with a roughness of 7 nm. After its functionalization with Py/Py-COOH/MNPs, the topography of the surface totally changed, presenting a homogeneous morphology with a roughness of 8.4 nm. The surface showed the presence of spherical shapes that indicated the presence of MNPs coated with Py/Py-COOH.

**Figure 3:**
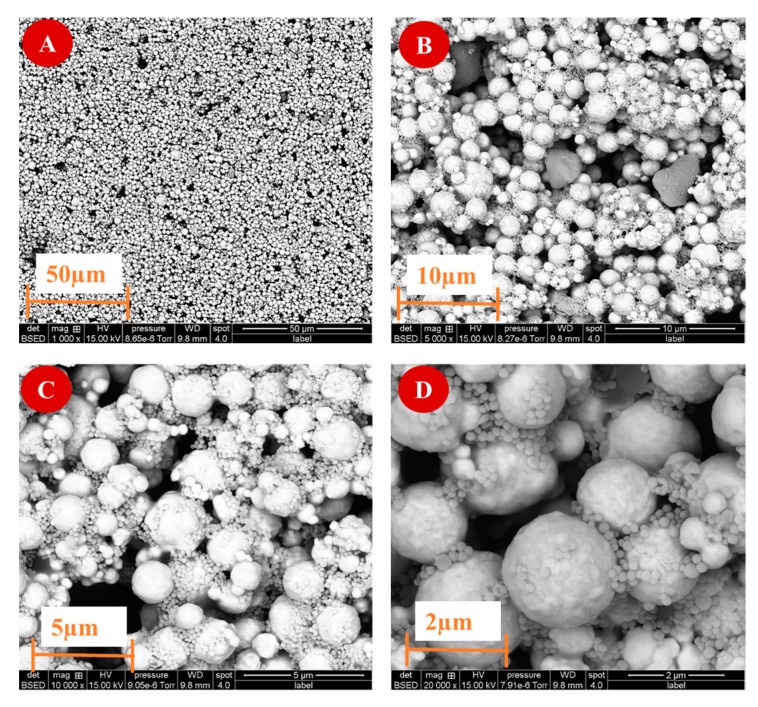
SEM images of Py/Py-COOH/MNPs electrodeposited onto SPEAu surface. (**A**) Label: 50 µm, mag: 1000×; (**B**) Label: 10 µm, mag: 5000×; (**C**) Label: 5 µm, mag: 10,000×; (**D**) Label: 2 µm, mag: 20,000×.

The morphology of Py/Py-COOH/MNPs electrodeposited onto the SPEAu surface was observed by SEM. As seen in Figure 3, MNPs are not smooth, and the surface roughness can be attributed to local surface polymerization.

### 3.2. Voltammetric Studies

**Figure 4:**
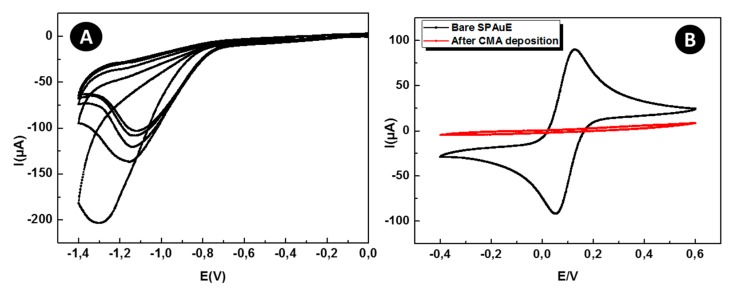
(**A**) Cyclic voltammogram of CMA deposition onto SPEAu. The black voltammogram corresponds to the first CV scan, whereas the magenta one to the last CV scan. (**B**) Cyclic voltammograms of bare SPEAu (black) and after CMA deposition (magenta) performed by immerging the device into a 5 mM K_3_[Fe(CN)_6_]/K_4_[Fe(CN)_6_] in a PBS (pH 7.4) solution as redox couple.

CV was used for CMA electrodeposition and to confirm the electrode surface complete coverage. As shown in Figure 4A, the initial cycle of CV showed a broad and irreversible cathodic wave with a peak potential at –1.3 V. This result indicates the diazoted CMA molecules attachment onto the gold surface by the diazonium salt reduction. The cathodic current was remarkably weakened in the successive CV scans, similarly to those reported for gold electrodes, as already described by Baraket [24,25]. Figure 4B shows the surface behavior before and after the functionalization. It can be seen that the oxidation-reduction peaks of the redox couple on bare SPEAu have totally disappeared after CMA deposition. This can be explained by the weak electron transfer kinetics of K_3_[Fe(CN)_6_]/K_4_[Fe(CN)_6_] caused by the CMA blocking layer.

**Figure 5:**
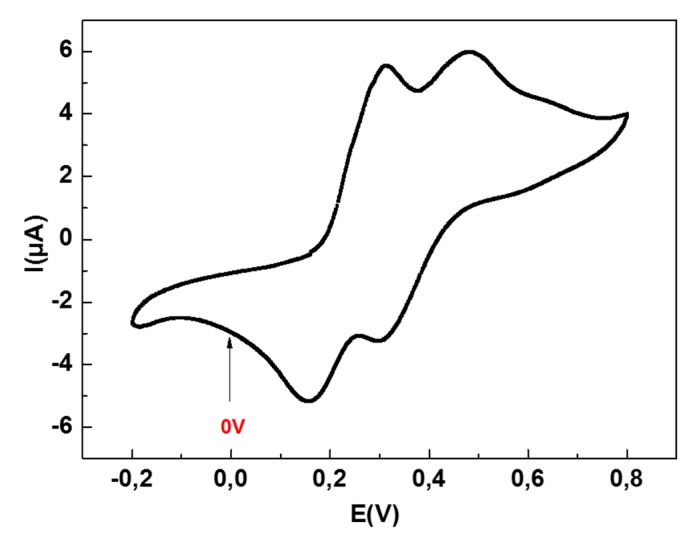
Cyclic voltammogram (CV) of bare screen printed gold SPEAu in TMB substrate (concentration not specified by the provider) at scan rate 100 mV·s^−1^. The presence of two oxidation and reduction peaks corresponds to the known two-electron redox behavior of TMB.

The CV technique was also used to investigate the behavior of TMB [26] with a SPEAu electrode. As shown in Figure 5, two pairs of peaks (Ep_OX1_ = +0.30 V, Ep_OX2_ = +0.48 V, Ep_RED1_ = +0.32 V, and Ep_RED2_ = +0.157 V, respectively) are present, which correspond to two reversible one-electron couples of TMB, observed at the bare gold electrode. In the presence of a peroxidase such as HRP, the oxidation of TMB is catalyzed, and the subsequent reduction can be recorded by applying a fixed voltage. The potential chosen was 0 V vs. Ag/AgCl. This potential was not chosen at the oxidation peaks so that the current change would not be due to the oxidation of any solution of TMB in the cell, nor to the reduction peaks, because the TMB is already reduced. In this way, we ensured that the current change in each chronoamperometric measurement was the result of the oxidation between the TMB substrate and the antibody conjugate Ab-TNF-α-HRP.

### 3.3. Detection of TNF-α in Standard Solution and Interference Study

**Figure 6:**
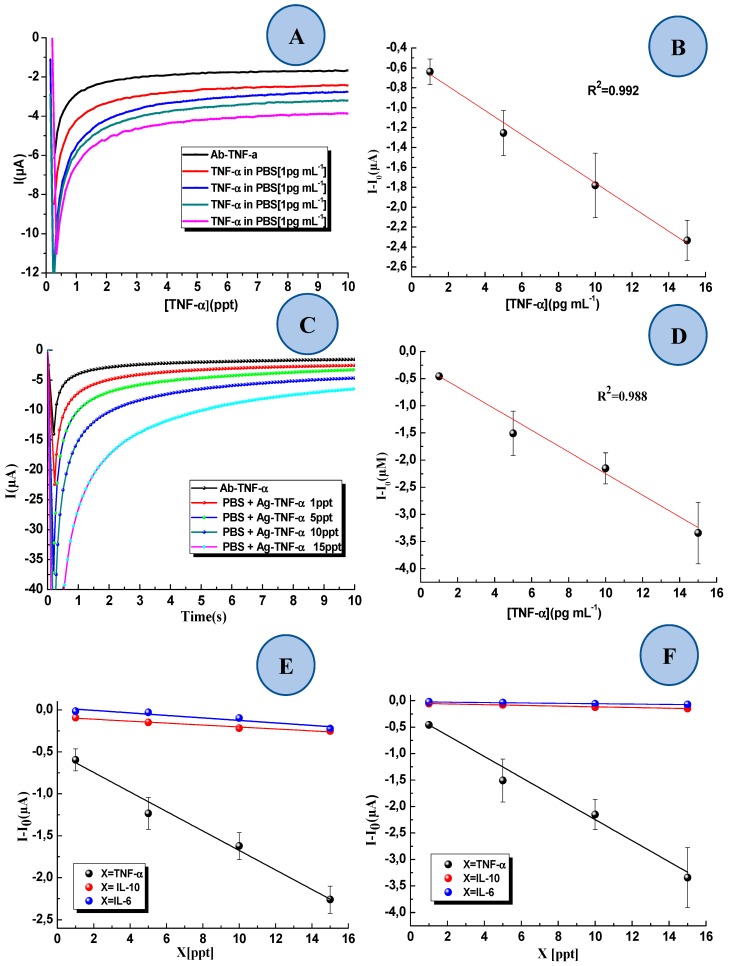
(**A**) Example of chronoamperometric responses of the 2D-SPEAu at 0 V (I vs. Time) of the immunosensor by analyzing TNF-α standard solutions with different concentrations (0, 1, 5, 10 and 15 pg·mL^−1^). (**B**) Sensitivity curve of the 2D-SPEAu immunosensor obtained by linear fitting of current values recorded after 10 s for the detection of Ag-TNF-α recorded for 10 s (y = ‒0.1116x − 2.019, R^2^ = 0.8423). Each TNF-α standard solution was analyzed in triplicate. (**C**) Example of chronoamperometric responses of the 3D-SPEAu at 0 V (I vs. Time) of the immunosensor by analyzing TNF-α standard solutions with different concentrations (0, 1, 5, 10 and 15 pg·mL^−1^). (**D**) Sensitivity curve of the 3D-SPEAu immunosensor obtained by linear fitting of current values recorded after 10 s for the detection of Ag-TNF-α recorded for 10 s (y = ‒0.1989x ‒ 0.2575, R^2^ = 0.9880). Each TNF-α standard solution was analyzed in triplicate. (**E**) Sensitivity curves related to the detection of (●) TNF-α, (●) IL-10, e and (●) IL-6 with 2D-SPEAu. (**F**) Sensitivity curves related to the detection of (●) TNF-α, (●) IL-10, e and (●) IL-6 with 3D-SPEAu.

Figure 6A,C show the chronoamperometric response of the two-dimensional and three-dimensional device, respectively. The black line represents the base line obtained in the blank sample (absence of TNF-α). The data indicates that both devices, i.e., with 2D-SPEAu or 3D-SPEAu, are capable of detecting TNF-α in a linear range from 1 to 15 pg·mL^−1^, but the immunosensor with the 3D-SPEAu showed a better linearity, with an R2 of 0.992. Moreover, the device precision, expressed as CV% on triplicate measurement for each concentration level, resulted in a 22% working rate with the 2D-SPEAu, whereas it was 8% with the 3D-SPEAu. LoD resulted 0.3 pg·mL^−1^ for both devices.

To study the sensitivity of both 2D-SPEAu and 3D-SPEAu devices, the immunosensor was tested by analyzing standard solutions containing possible interferences, such as IL-6 and IL-10. These analytes were chosen because they may represent other cytokines secreted in the acute stages of inflammation. As seen in Figure 6E,F, the 3D-SPEAu immunosensor showed a higher selectivity to TNF-α compared to IL-6 and IL-10. The detection of TNF-α demonstrates linearity upon increasing the concentration, with a sensitivity of –0.116 µA·ppt^−1^, but significant reduced current values were found by analyzing IL-6 (with a sensitivity of –0.0149 µA·pg·mL^−1^) and IL-10 (with a sensitivity of −0.0116 µA·pg·mL^−1^) standard solutions. All the equations and correlation coefficients (R^2^) of the obtained calibration curves are reported in Table 1.

Table 2. summaries the performances of 2D- and 3D-SPEAu. Due to higher linearity, precision and sensitivity, 3D-SPEAu was chosen for the detection of TNF-α in AS.

Table 3 lists a comparison of LoD reached with different biosensors reported in the literature to measure TNF-α in both artificial and human saliva samples. The linear range investigated is not the largest, but this would be irrelevant, as it is possible to dilute the sample, as demonstrated by performing the standard addition.

### 3.4. Detection TNF-α in Artificial Saliva with the 3D-SPEAu Immunosensor

**Figure 7:**
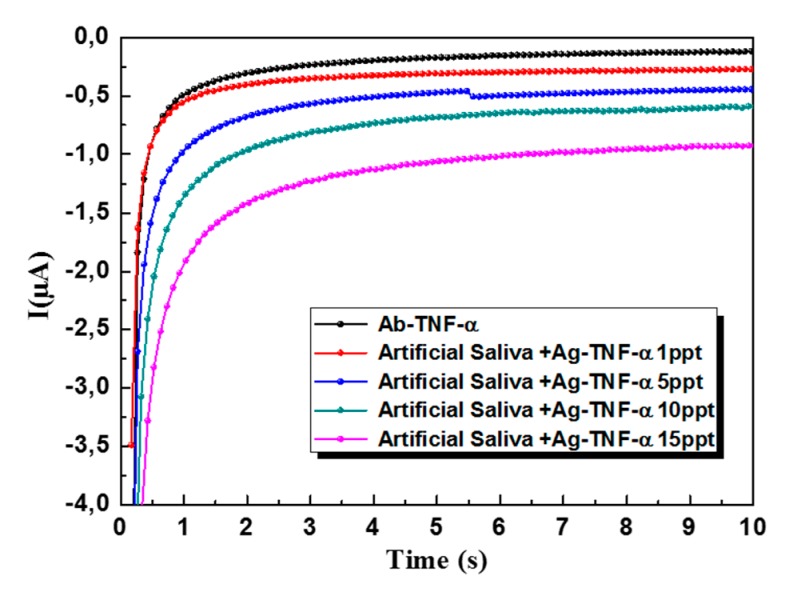
Chronoamperometric responses of the 3D-SPEAu at 0 V (I vs. Time) by analyzing AS samples spiked with different concentrations of TNF-α (0, 1, 5, 10 and 15 pg·mL^−1^).

The linear response of the 3D-SPEAu immunosensor was investigated also by analyzing AS samples spiked with increasing TNF-α concentration. Figure 7 shows the chronoamperometric responses at 0 V (I vs. Time) obtained after subsequently incubations in AS samples containing different concentrations of TNF-α (0, 1, 5, 10 and 15 pg·mL^−1^). Linear fitting of current values recorded after 10 s gave y = −0.0313x − 0.0968 as the equation of the corresponding calibration curve, with an R^2^ of 0.998.

Finally, three AS samples spiked with a known amount of TNF-α (5 pg·mL^−1^) were analyzed to simulate the 3D-SPEAu application on a real sample performing the standard additions method as already described in Section 2.7. Taking care of the dilution factor in the sample preparation, the mean concentration in the sample was 5.1 ± 0.8 pg·mL^−1^, which was in complete agreement with the expected result of 5.0 pg·mL^−1^.

## 4. Conclusions

The aim of this work was the development of a new immunosensor for the detection of TNF-α in saliva samples by chronoamperometry. The immunosensor is based on SPEAu, and two different functionalization strategies have been designed and compared. A 2D-SPEAu was developed by chemical bonding of Ab-TNF-α onto the electrode surface, whereas the 3D-SPEAu was obtained by electrodeposition of Py/Py-COOH/MNPs by PCA. Due to the superior conductivity and larger surface area, the 3D-SPEAu electrode resulted in a more sensitive device, showing very good performance and sensitivity for the detection of TNF-α, even in presence of other possible interference such as by other inflammatory cytokines (e.g., IL-6 and IL-10). The immunosensor with the 3D-SPEAu allowed us to obtain very promising results also in analyzing AS samples spiked with TNF-α. Thus, further implementations of the proposed immunosensor would make it possible to obtain a useful tool for the diagnosis and monitoring of HF by saliva analysis.

## Figures and Tables

**Table 1:** Equations of calibration curves and related correlation coefficients obtained by analyzing standard solution of TNF-α, IL-6 and IL-10 using both 2D- and 3D-SPEAu.

Standard Solution Containing	Calibration Curve (R^2^)	
	2D-SPEAu	3D-SPEAu
**TNF-α**	y = –0.1161x – 0.5130 (0.984)	y = –0.1990x – 0.2575 (0.988)
**IL-6**	y = –0.0149x – 0.0242 (0.873)	y = –0.0036x – 0.0205 (0.971)
**IL-10**	y = –0.0161x – 0.0876 (0.967)	y = –0.0068x – 0.0476 (0.974)

**Table 2:** Comparison of 2D-SPEAu and 3D-SPEAu performances.

Immunosensor with	R^2^	LoD [pg·mL^−1^]	CV%	Sensitivity (µA pg·mL^−1^)
2D-SPEAu	0.8423	0.3	22	0.116
3D-SPEAu	0.9812	0.3	8	0.199

**Table 3:** Limit of detection (LoD) of different biosensors recently used for TNF-α determination in artificial or human saliva.

Technique	Linear Range	LoD	Reference
Impedance spectroscopy	1–100 pg·mL^−1^	1 pg·mL^−1^	[23]
Impedance spectroscopy	0.01–2 pg·mL^−1^	3.7 fg·mL^−1^	[27]
Amperometry	1–200 pg·mL^−1^	0.85 pg·mL^−1^	[28]
Amperometry	1–30 pg·mL^−1^	1 pg·mL^−1^	[22]
Amperometry	1–15 pg·mL^−1^	0.3 pg·mL^−1^	This work
